# Daily skin-to-skin contact alters microbiota development in healthy full-term infants

**DOI:** 10.1080/19490976.2023.2295403

**Published:** 2024-01-10

**Authors:** Henrik Andreas Eckermann, Jennifer Meijer, Kelly Cooijmans, Leo Lahti, Carolina de Weerth

**Affiliations:** a Donders Institute for Brain, Cognition and Behaviour, Department of Cognitive Neuroscience, Radboud university medical center, Nijmegen, The Netherlands; b Behavioural Science Institute, Radboud University, Nijmegen, The Netherlands; c Department of Computing, University of Turku, Turku, Finland

**Keywords:** Gut microbiota development, skin-to-skin intervention, early development, infancy, RCT

## Abstract

The gut microbiota is vital for human body development and function. Its development in early life is influenced by various environmental factors. In this randomized controlled trial, the gut microbiota was obtained as a secondary outcome measure in a study on the effects of one hour of daily skin-to-skin contact (SSC) for five weeks in healthy full-term infants. Specifically, we studied the effects on alpha/beta diversity, volatility, microbiota maturation, and bacterial and gut-brain-axis-related functional abundances in microbiota assessed thrice in the first year. Pregnant Dutch women (*n* = 116) were randomly assigned to the SSC or care-as-usual groups. The SSC group participants engaged in one hour of daily SSC from birth to five weeks of age. Stool samples were collected at two, five, and 52 weeks and the V4 region was sequenced. We observed significant differences in the microbiota composition, bacterial abundances, and predicted functional pathways between the groups. The SSC group exhibited lower microbiota volatility during early infancy. Microbiota maturation was slower in the SSC group during the first year and our results suggested that breastfeeding duration may have partially mediated this relation. Our findings provide evidence that postpartum SSC may influence microbiota development. Replication is necessary to validate and generalize these results. Future studies should include direct stress measurements and extend microbiota sampling beyond the first year to investigate stress as a mechanism and research SSC’s impact on long-term microbiota maturation trajectories.

## Introduction

The human gastrointestinal tract is inhabited by a complex population of bacteria. These bacteria allow the digestion of dietary fibers, providing absorption of nutrients and energy.^1^ They play an important role in intestinal integrity and immune functioning.^2,3^ In addition, gut bacteria can influence the brain via the gut-brain axis, a bidirectional pathway between the gut and the brain.^4,5^ The complex mechanisms underlying this bidirectional communication are still subject of study and have been thoroughly summarized elsewhere.^4^ Briefly, the gut microbiota can influence human physical and mental development via the immune system, tryptophan metabolism, the vagus nerve and the enteric nervous system. This communication involves microbial metabolites such as short-chain fatty acids, branched chain amino acids and peptidoglycans.

During the early stages of life, the gut microbiota and other co-evolving systems are particularly sensitive to environmental disturbances. Furthermore, the establishment of a healthy gut microbiota during early life is important for the functioning of other systems, such as the immune system.^6,7^ Therefore, it is important to understand how the gut microbiota develops and how it is influenced during infancy. The present study investigated the potential effects of a randomized controlled trial (RCT) involving daily skin-to-skin contact (SSC) between mothers and their full-term infants on the developing gut microbiome.

The fetal gut is thought to be virtually sterile, although the sterility of the intrauterine environment and meconium is still subject of debate.^8^ Starting from birth, the mother is the infant’s main source of microbial gut colonization via vaginal delivery, breastfeeding, and frequent close contact. This transmission of bacteria will remain detectable even at older ages.^9–11^ Subsequently, other household members, close contacts, and pets may become sources of bacteria.^11,12^ The infant’s gut microbiota starts to increase in diversity and develops toward an adult-like microbiota as solid foods are introduced and breastfeeding is cessated,^13^ although important changes are still seen in middle childhood.^14^ Finally, infant health, use of antibiotics, hygiene, and infant genetics are important factors that contribute to the development of the gut microbiome.^9,15^

During SSC, the naked infant, dressed only in a diaper, is placed on the bare chest of the mother.^16^ SSC can be considered de-stressing^17–19^ and has been shown to be beneficial for both mother and infant. In preterm infants, SSC immediately after birth is associated with improved health outcomes, as well as a reduced mortality rate for the infant, and improved caregiving behavior and lower postpartum depression for the mother.^20–29^ After the first postnatal hours, daily SSC with pre-terms is associated with better physical outcomes and improved development of the brain and the cardiovascular system.^22,30,31^ Interestingly, the effect of daily SSC on cognitive function in pre-terms was still seen in young adulthood.^32,33^ While the focus of these studies remains on preterm infants, some studies have investigated full-term infants, although these studies are mostly limited to SSC in the first hours after birth. They have indicated several benefits of SSC right after birth, such as improved cardiovascular health, improved sleep, and weight gain.^19,29^ Concerning the mother, SSC performed on full-term infants right after birth is associated with a decrease in anxiety and longer breastfeeding duration.^29,34,35^ Prolonged, daily SSC is associated with a reduction in maternal depressive symptoms,^36^ anxiety and stress.^37^ The SKIPPY study, of which this current study is part, was the first to perform an RCT to study an SSC intervention on both maternal and infant outcomes in a healthy, full-term sample.^38^ The study showed that performing a daily hour of SSC during the first five postnatal weeks may reduce maternal anxiety and fatigue symptoms, increase infant sleep and reduce infant crying, and extend exclusive and continued breastfeeding durations.^39–41^ At three years of age, the children that had received the SSC intervention also showed fewer internalizing and externalizing behavioral problems.^42^ However, the results did not indicate that this daily hour of SSC influenced mother-infant interaction quality and maternal depressive, stress, and pain symptoms, as was found in earlier intervention studies.^39,43^

There are multiple pathways in which mother-infant SSC could potentially influence the infant gut microbiome. Firstly, as mentioned before, a daily hour of SSC was associated with fewer maternal anxiety and fatigue symptoms.^39^ Previous studies have indicated a link between maternal postnatal distress (including anxiety) and breast milk microbiota, which in turn could influence the gut microbiome of breastfed infants.^44,45^ Secondly, breastfeeding shapes the infant gut microbiome.^46^ The extension of exclusive and continued breastfeeding duration caused by a daily hour of SSC could therefore influence the infant gut microbiome. Third, SSC may also be de-stressing for infants.^17–19^ Stress during the early developmental stages has been found to influence the gut microbiota and gut-brain axis in rodents.^47,48^ Therefore, a de-stressing practice such as SSC could potentially alter the colonization of the gut.^49–51^ Lastly, SSC could also provide an additional opportunity for the exchange of microbes between the mother and child. Previous studies have shown that constant contact between microbial communities increases their similarity^52,53^ and the maternal skin microbiome has been shown to provide a source of bacteria for infants.^54,55^

To the best of our knowledge, there have been no previous studies on the effects of SSC on the infant gut microbiome. The current study investigated the effects of a five-week daily hour SSC between mothers and full-term infants, compared to care-as-usual (CAU), on the infant gut microbiome. We hypothesized that the treatment and the control group differ in (1) alpha diversity, (2) beta diversity, (3) genus level abundances, (4) volatility, (5) microbiota age and (6) functional pathways that are related to gut-brain communication. While most hypotheses are non-directional, we hypothesized that SSC infants have less volatile microbiota, since stress has been shown to increase gut microbiota volatility.^56^

## Results

### Descriptive statistics

**Figure 1.**
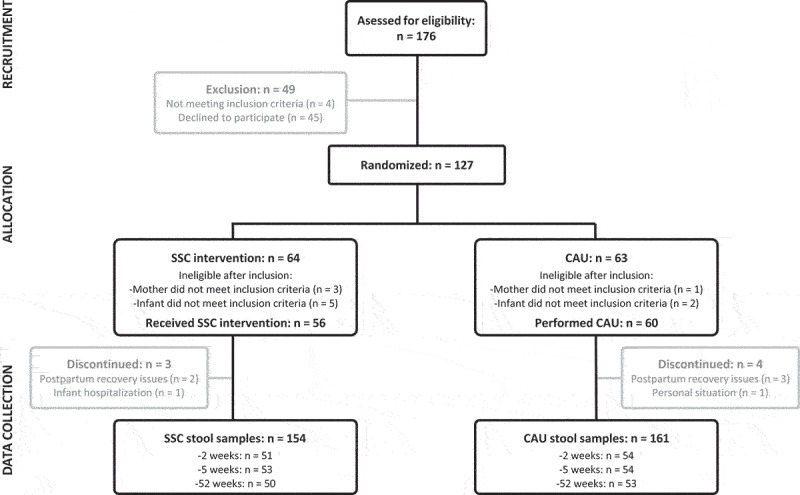
Participant flow diagram, including the number of participants at each of the trial stages. SSC = skin-to-skin contact. CAU = care-as-usual.

**Figure 2.**
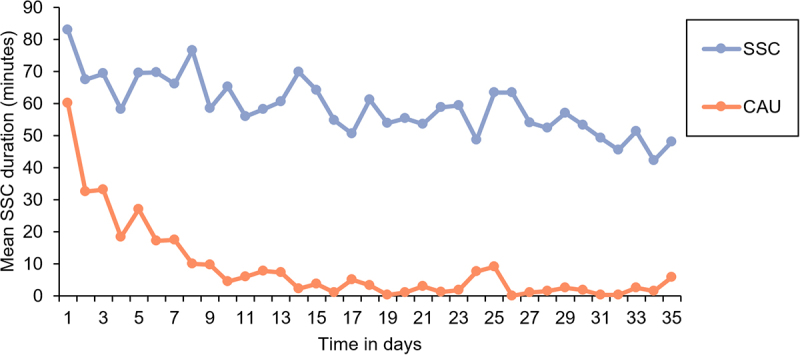
Mean daily skin-to-skin contact (SSC) duration in the SSC and care-as-usual (CAU) condition based on data derived from the intention-to-treat selection.

Participation in stool sample collection and eligibility resulted in 116 participants (Figure 1). For one stool sample, PCR amplification did not provide enough material for sequencing. While there was no loss to follow-up in this RCT, some mothers did not provide stool samples for all time points leading to a total of 315 analyzed samples: 105 at week two, 107 at week five and 103 at week 52. Across all time points, 11 phylum-level groups and 162 genus-level groups were identified. The SSC group provided longer total SSC duration (intention-to-treat (ITT): 2067.67 ± 850.65 min; per protocol (PP): 2905.90 ± 497.52 min) than the CAU group (ITT: 308.17 ± 442.41; PP: 308.17 ± 442.41) according to independent sample t-tests (*p* < 0.001). The average daily SSC duration between groups is furthermore depicted in Figure 2. An overview of the baseline characteristics of the infants and their mothers, including information on missing data, is presented in Table 1. In the following, we present for each preregistered feature of the microbiota the results of the ITT analysis and in case of meaningful deviation, the PP analysis. Relevant parameter estimates, such as β-coefficients of the Bayesian robust linear models, are mentioned in the text. To inspect all model coefficients, see supplementary Figures S1–6.

**Table 1.** Descriptive statistics and group comparisons of raw data for mother – infant dyads of the SKPPY study.

	CAU	SSC	Overall
	(*N* = 60)	(*N* = 56)	(*N* = 116)
Sex			
Male	26 (43.3%)	33 (58.9%)	59 (50.9%)
Female	34 (56.7%)	23 (41.1%)	57 (49.1%)
C-section			
No	55 (91.7%)	51 (91.1%)	106 (91.4%)
Yes	3 (5.0%)	4 (7.1%)	7 (6.0%)
Missing	2 (3.3%)	1 (1.8%)	3 (2.6%)
Gestational age (weeks)			
Mean (SD)	40.0 (1.10)	40.1 (1.01)	40.0 (1.05)
Median [Min, Max]	40.2 [37.1, 42.1]	40.2 [36.6, 42.1]	40.2 [36.6, 42.1]
Birth weight (grams)			
Mean (SD)	3570 (386)	3650 (415)	3610 (401)
Median [Min, Max]	3520 [2880, 4620]	3670 [2740, 4850]	3610 [2740, 4850]
Siblings			
No	28 (46.7%)	27 (48.2%)	55 (47.4%)
Yes	32 (53.3%)	29 (51.8%)	61 (52.6%)
Exclusive breastfeeding duration (months)			
Mean (SD)	2.78 (1.47)	3.14 (1.86)	2.96 (1.68)
Median [Min, Max]	3.00 [0, 5.00]	3.00 [0, 9.00]	3.00 [0, 9.00]
Missing	9 (15.0%)	7 (12.5%)	16 (13.8%)
Age week 2 (days)			
Mean (SD)	14.9 (1.25)	14.1 (1.60)	14.5 (1.49)
Median [Min, Max]	14.0 [14.0, 19.0]	14.0 [8.00, 16.0]	14.0 [8.00, 19.0]
Missing	13 (21.7%)	8 (14.3%)	21 (18.1%)
Age week 5 (days)			
Mean (SD)	35.9 (2.33)	35.1 (1.88)	35.5 (2.13)
Median [Min, Max]	35.0 [28.0, 40.0]	35.0 [28.0, 39.0]	35.0 [28.0, 40.0]
Missing	13 (21.7%)	7 (12.5%)	20 (17.2%)
Age week 52 (days)			
Mean (SD)	371 (7.56)	370 (7.89)	371 (7.70)
Median [Min, Max]	369 [364, 399]	368 [362, 406]	368 [362, 406]
Missing	9 (15.0%)	9 (16.1%)	18 15.5%)
Antibiotics week 2			
No	47 (78.3%)	43 (76.8%)	90 (77.6%)
Yes	1 (1.7%)	2 (3.6%)	3 (2.6%)
Missing	12 (20.0%)	11 (19.6%)	23 (19.8%)
Antibiotics week 5			
No	46 (76.7%)	42 (75.0%)	88 (75.9%)
Yes	2 (3.3%)	1 (1.8%)	3 (2.6%)
Missing	12 (20.0%)	13 (23.2%)	25 (21.5%)
Antibiotics week 52			
No	50 (83.3%)	46 (82.1%)	96 (82.8%)
Yes	1 (1.7%)	1 (1.8%)	2 (1.7%)
Missing	9 (15.0%)	9 (16.1%)	18 (15.5%)

## Main results

### Alpha diversity

**Figure 3.**
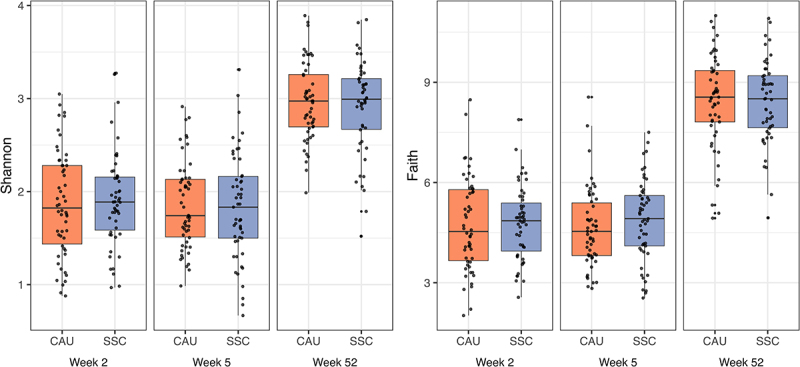
Alpha diversity indices between treatment (SSC) and control (CAU) group per sampling time point.

Our results indicated that SSC had no effect on alpha diversity across different alpha diversity indices, time points, and ITT and PP analyses (Figure 3). Infants with siblings had lower Shannon alpha diversity scores (β = −0.31, 95% HDI [−0.52;-0.09], $P\left(\beta <0\right)$ = 0.997) in early infancy (2 and 5 week samples), but no longer in late infancy (1 year samples). Results regarding siblings were similar for Chao1 and the inverse Simpson, but the difference disappeared mostly when the Faith index was used.

### Beta diversity

**Figure 4.**
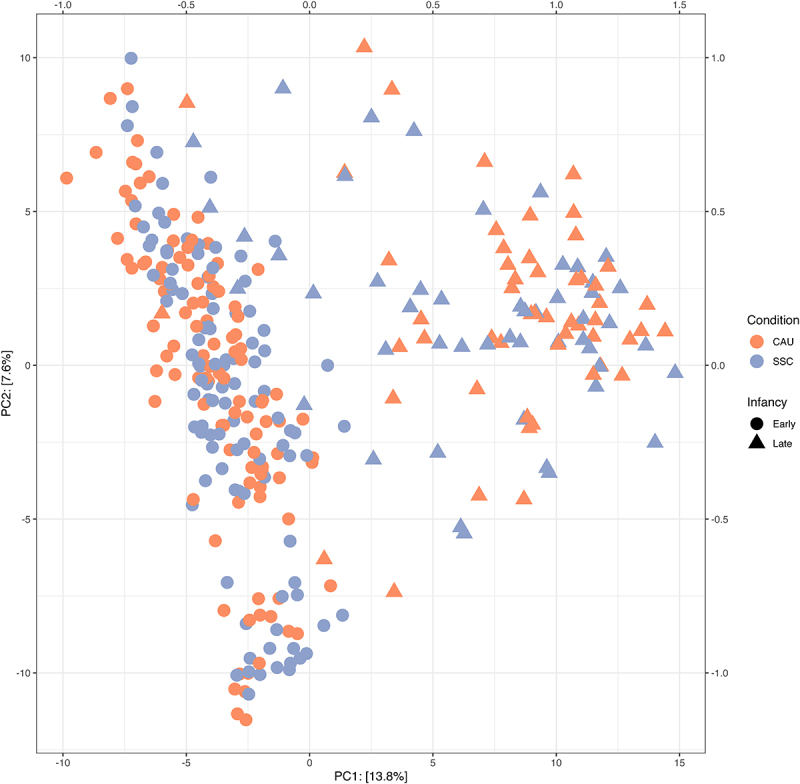
All samples plotted on the first 2 dimensions of a PCA of Euclidean distances of CLR transformed abundance values (Aitchison distance).

Figure 4 shows the results of a PCA of the centered-log-ratio transformed abundances (Aitchison distance). Samples obtained at one year (triangles) were clearly separated from samples obtained at two and five weeks. There seems to be no apparent separation of SSC and CAU samples considering all samples, also not when other principal components were inspected (not shown here). Within the 1-year samples (triangles), the SSC samples (gray) may occur more frequently closer to the early infancy samples than the CAU samples. Statistically, PERMANOVA identified that the microbiota composition of CAU and SSC samples differed significantly in the early infancy samples (*p* = 0.016), but not in the late infancy samples (*p* = .087). To derive the direct (rather than the total) effect of SSC under the assumed directed acyclic graph (Figure S9), we fitted new models including breastfeeding as a covariate. The effect remained significant, and the effect size unchanged, suggesting that SSC has an effect on microbiota composition independent of its effect on breastfeeding duration. In the PP analyses, the effect size increased slightly, but the standard error also increased because of the lower sample size, leading to a non-significant effect (*p* = .058). Note also that the result was sensitive to the choice of another distance metric. When using Bray Curtis similarity, the main effect was no longer significant (*p* = .377) while the interaction term suggested that SSC may have only had an effect on the microbiota at 5 weeks (*p* = .069). Using Bray Curtis similarity, SSC only showed a significant effect in the PP analyses when fitting a model to all samples (*p* = .035).

### Genus level abundances

**Figure 5.**
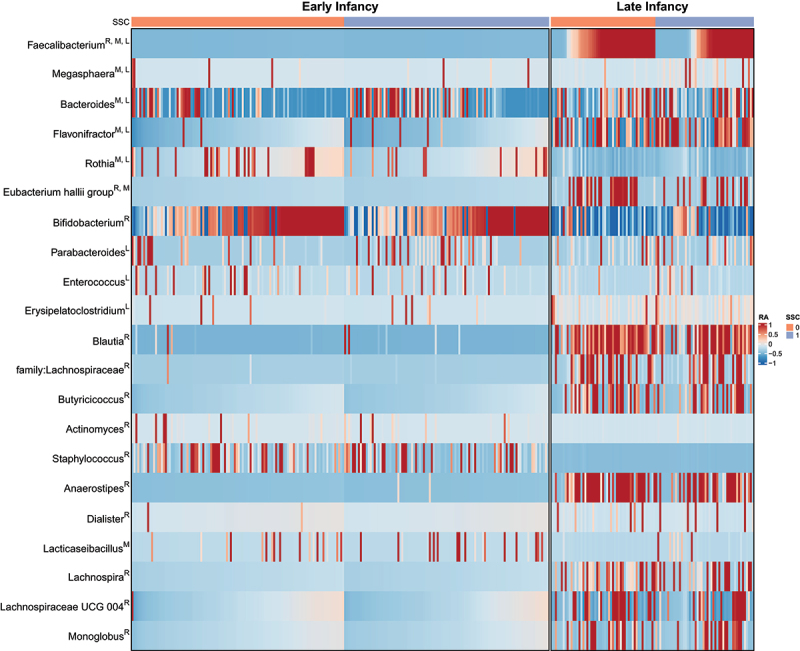
Heatmap of clr-transformed bacterial abundances that were either differentially abundant between treatment (SSC) and control (CAU) or important for the microbiota age model. The letter behind each genus indicates whether it was important for the microbiota age model (R, see corresponding section) or identified by any of the differential abundance analysis methods (*M* = Maaslin2, L = LinDA). The relative abundance values have been scaled to zero mean and unit variance in order to highlight differences in the variation relative to the mean level within each taxonomic group. The color scale has been limited to the interval [−1, 1].

Because recent benchmark studies have shown that differential abundance analysis results can depend heavily on the specific method that is applied,^57,58^ we used several methods to evaluate the robustness of findings across methods. While we focus on the results of MaAsLin2 in this section, Figure 5 indicates whether a taxon was identified by one or more methods (denoted as M (MaAsLin2), L (LinDA) or A (ANCOMBC), respectively). MaAsLin2 detected a lower relative abundance of *Faecalibacterium*, *Eubacterium hallii*, and *Rothia* and higher abundance of *Flavonifractor*, *Lacticaseibacillus*, *Bacteroides* and *Megasphaera* in the SSC group compared to the CAU group. The top six genera in Figure 5 stand out, as they were identified as drivers of differences between groups in at least two methods. From the heatmap, we can further infer that some genera were only differentially abundant in either early or late infancy. Note that ANCOMBC, the most conservative method, did not detect any genera that were differentially abundant.

### Microbiota volatility

**Figure 6.**
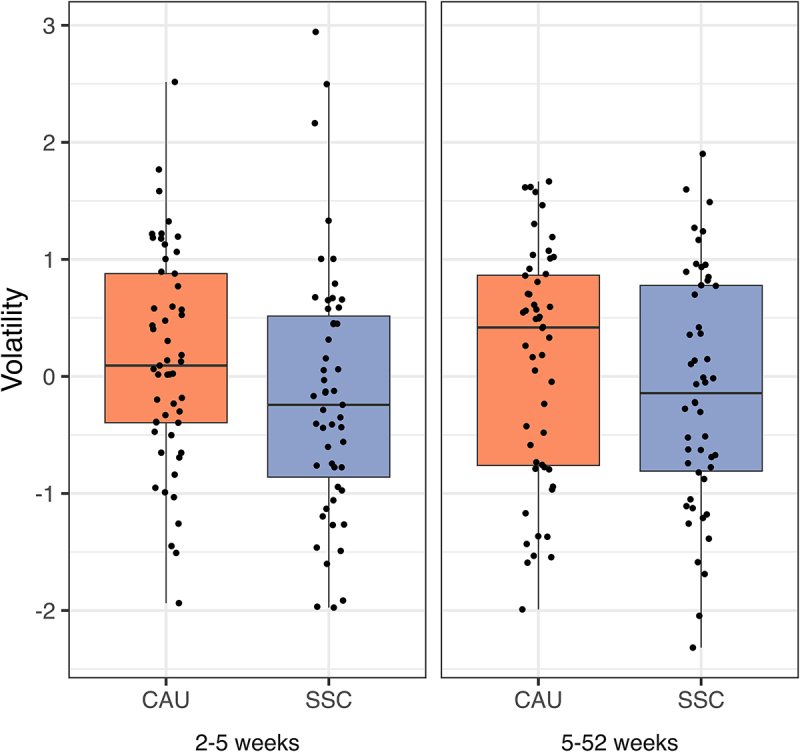
Volatility (intra-individual Aitchison distance) between treatment (SSC) and control (CAU) group for the sample sequence from 2 to 5 weeks and 5 to 52 weeks.

Microbiota volatility is defined as the intra-individual change in microbiota composition over time and was calculated as described by Bastiaanssen et al.^59^ We fitted Bayesian robust linear models to the volatility scores in early infancy (2–5 weeks) and from early to late infancy (5–52 weeks) (Figure 6). Volatility was lower in the SSC group in early infancy than in the CAU group (β = −0.31, 95% HDI [−0.62;0], $P\left(\beta <0\right)$ = 0.95), rejecting the null hypothesis that SSC has no influence on microbiota volatility. This effect remained unchanged after including breastfeeding in the regression model, suggesting that SSC affects microbiota volatility in early infancy, independent of its effect on breastfeeding. Note that in the PP analyses for early infancy, the effect size increased slightly, whereas the highest density interval widened with a smaller sample size(β = −0.4, 95% HDI [−0.88;0.09], $P\left(\beta <0\right)$ = 0.92). While average volatility was also lower in the SSC group when looking at the distances between the 5 and 52 weeks samples, the effect did not pass the decision criterion (β = −0.16, 95% HDI [−0.47;0.15], $P\left(\beta <0\right)$ = 0.80) to reject the null hypothesis. Breastfeeding (β = −0.13, 95% HDI [−0.22;-0.03], $P\left(\beta <0\right)$ = 0.99) and gestational age (β = −0.25, 95% HDI [−0.46;-0.04], $P\left(\beta <0\right)$ = 0.99) were negatively related to volatility (breastfeeding only for volatility between 5 and 52 weeks).

### Microbiota age

**Figure 7.**
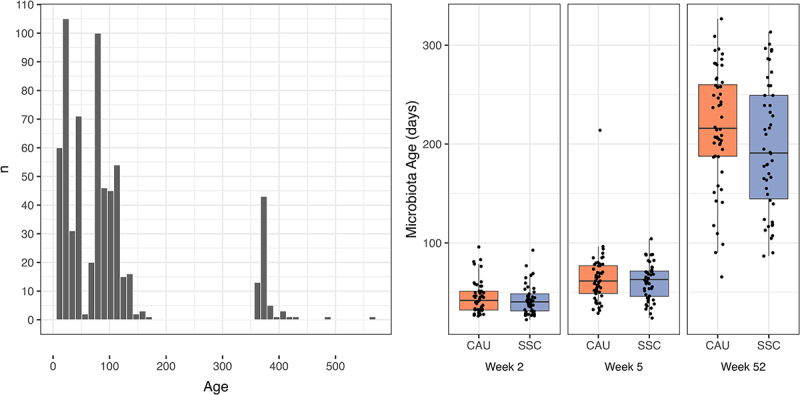
A. Age of sample collection for the samples used to train the Random Forest model. B. Predicted microbiota age for the samples analyzed in this study between treatment (SSC) and control (CAU) per time point.

Microbiota age was estimated as described by Subramanian et al. by training a Random Forest model using samples from two other Dutch longitudinal studies (BIBO, BINGO) in the age range of 7–561 days (Figure 7A).^60^ The Random Forest model performance was comparable to that of Subramanian et al.^60^ as 64% of variance in age could be explained by the microbiota. We evaluated the model fit and significance by computing the correlation between predictions and actual ages using SKIPPY samples (*r* = 0.862, *p* < 0.001). Besides illustrating the results of the differential abundance analysis, Figure 5 shows the most important features of the RF model (top 15 with non-zero abundance in the SKIPPY samples; denoted by R next to the taxon name in Figure 5). To predict microbiota age, *Faecalibacterium* was the most informative next to other genera that are known to either dominate early infancy due to breastfeeding (*Staphylococcus* and *Bifidobacterium*) or to only occur with the cessation of breastfeeding and introduction of solid food as dominance of *Bifidobacterium* disappears. Note that we have lower coverage of the ages around 1 year in BIBO and BINGO compared to the coverage of ages in early infancy (Figure 7A), resulting in higher uncertainty in the estimation of the microbiota age reference compared to the time period early in infancy. Nevertheless, we could compare the microbiota ages of the treatment and control groups in the SKIPPY cohort. Figure 7B depicts the microbiota age scores at each time point between the treatment groups in the SKIPPY samples. The samples obtained at one year show relatively low microbiota age, indicating that the SKIPPY cohort had lower microbiota ages than the BIBO and BINGO cohorts. The Bayesian robust regression models indicated that treatment was associated with a lower microbiota age with an average decrease of 25.65 days (β = −25.65, 95% HDI [−50.37;-1.31], $P\left(\beta <0\right)$ = 0.98) at one year of age. After adding breastfeeding, the effect size decreased (β = −19.32, 95% HDI [−47.90;8.01], $P\left(\beta <0\right)$ = 0.90)) while breastfeeding was associated with lower microbiota age, as expected (β = −7.27, 95% HDI [−13.94;-0.62], $P\left(\beta <0\right)$ = 0.98). These results indicate that the SSC intervention influenced microbiota development with visible effects at 1 year of age. Breastfeeding duration may have partially mediated this effect. An exploratory dose-response analysis within only the treatment group indicated that there was no stronger effect on microbiota age when subjects provided more hours of SSC. Thus, infants in the treatment group were probably mostly above the minimal number of hours needed to produce the observed effect on microbiota age (Figure S7).

### Gut brain module abundances

We estimated 44 metabolic pathways related to gut-brain communication (gut brain modules).^61^ These pathways include, for example, serotonin degradation or histamine production. Using MaAsLin2 (FDR≤0.2), we found that nitric oxide degradation was lower (FDR = 0.166), while butyrate synthesis I (FDR = 0.169) and acetate synthesis III (FDR = 0.148) were higher in the SSC group compared to the CAU group (Figure S8).

## Discussion

We hypothesized that a skin-to-skin intervention (SSC) applied in the first weeks of life in full-term infants would influence features of the gut microbiota via several potential mechanisms. These include physical transmission,^55^ prolonged breastfeeding^40^ and by having a stress-reducing, stabilizing effect on the developing infant.^17–19^ The microbiota features that were investigated included alpha-diversity, beta-diversity, genus level abundances, volatility, microbiota age and abundances of selected predicted functional pathways (gut brain modules; GBMs). The results provide evidence that a postpartum skin-to-skin intervention may influence gut microbiota development.

### Lower microbiota volatility in the SSC group

Infants that received the SSC intervention significantly differed in their overall microbiota composition and had lower microbiota volatility in early infancy. Volatility has been found to be higher in stressed mice, in humans reporting higher experienced stress during exam periods, and in patients with inflammatory bowel disease.^56,62^ In addition, a related metric indicated higher volatility (lower stability) in infants with colic.^63^ We furthermore observed that higher gestational age was associated with lower volatility in both time windows, possibly indicating that infants who are further in their physical development for their chronological age have more stable gut microbiota.

### Lower microbiota age in the SSC group

Infants in the SSC group had a lower microbiota age at one year of age compared with the control group. Our results further indicated that breastfeeding may have partially mediated this effect of SSC on microbiota age, which is in line with previous research showing that breastfeeding duration is negatively associated with microbiota maturation.^13,64^ However, after controlling for months of exclusive breastfeeding or alternatively, age at weaning, SSC infants still had, on average, 19 or 15 days, respectively, lower microbiota age. In addition to breastfeeding, lower microbiota age has been associated with exposure to antibiotics, delivery via cesarean section,^65^ and asthma.^64^ We found that siblings and educational level were positively associated with microbiota age at one year. The former is in line with previous research showing that infants with older siblings show faster maturation of the microbiota^66^ and specifically earlier colonization with *Faecalibacterium prausnitzii*.^67^ Colonization with *Faecalibacterium prausnitzii* is a characteristic of microbiota maturation^68^ and this taxon was the most important feature in our microbiota age model.

It is important to note that environmental factors appear to alter the dynamics of microbiota maturation rather than the speed of maturation. For example, formula-fed infants or infants born via cesarean section showed initially higher microbiota ages (in the first 6–12 months) before they started to show lower or similar microbiota ages compared to breastfed or vaginally born infants at later developmental stages.^65^ A reverse dynamic was observed for antibiotic exposure with initially lower and later temporarily higher microbiota age compared to individuals not exposed to antibiotics.^65^ The diverse associations with microbiota age raise the question of which specific aspects of microbiota maturation at which specific ages may be beneficial or possibly unfavorable for healthy development.

To investigate this further, it would be desirable for future research to utilize a common microbiota age model trained on large samples for the respective geographic region. Combined with sampling for a longer time span (e.g., the first three years), it may be possible to describe different trajectories of microbiota maturation that may be related to environmental variables, and in longitudinal follow-ups, to health outcomes in later life. Finally, while increasing alpha diversity is a characteristic of microbiota maturation, we did not observe any differences in alpha diversity between the groups. Our observation that siblings was associated with lower alpha diversity and with higher microbiota age might seem contrary, but note that having one or more siblings was only negatively associated with alpha diversity in early infancy and not at 1 year of age, in line with findings in another Dutch cohort^12^ as well as with those of other studies.^69–72^

### Differential abundance of individual genera between groups

Differential abundance analysis highlighted several genera (*Faecalibacterium*, *Megasphaera*, *Bacteroides*, *Flavonifractor*, *Rothia* and *Eubacterium hallii*) as well as predicted functional pathways (nitric oxide degradation, butyrate synthesis I, and acetate synthesis III) as differentially abundant between the groups. *Faecalibacterium prausnitzii* is well known as a butyrate producer, and because of that, and its negative relationship with inflammatory bowel diseases, it is generally considered a health-promoting species.^67,73^ Laursen et al.^67^ found that the prevalence was between approximately 55% and 80% at 9–10 months of age and increased to almost 100% at 16 months of age in three separate infant populations. The abundance of *Faecalibacterium* increased substantially after 1 year of age, but no longer from 3 to 5 years of age.^67,68^ In our study sample, the prevalence of *Faecalibacterium* at 12 months of age was 86.8% for the CAU and 68.0% for the SSC group. This finding, combined with the previously discussed findings of the microbiota age model, indicates a slower speed of microbiota maturation in the SSC group along a normal trajectory of microbiota development until the first year of life. This is supported by the fact that while the microbiota of infants typically matures along similar trajectories, differences in speed between individuals have been documented.^68^

In contrast to *Faecalibacterium*, the abundance of *Bacteroides* was higher in the SSC group. Higher abundance at 1 year of age has previously been associated with enhanced neurodevelopment.^74^ *Megasphaera* was more abundant in the SSC group. This genus includes butyrate- and propionate-producing strains^75^ and has been negatively associated with diarrheal cryptosporidiosis^76^ and maternal stress.^77^ However, the relationship with maternal stress was only present at 6 weeks and 3 months of age and reversed at 6 months of age. *Flavonifractor*, also higher in the SSC group, has been negatively associated with the development of asthma later in life^78^ and was able to strongly suppress Th2 immune responses in mice.^79^ Furthermore, *Flavonifractor plautii* was negatively associated with maternal stress in samples obtained at 6 weeks, 3 months, and 6 months.^77^ *Rothia*, with a slightly lower relative abundance in the SSC group, has been reported to be increased in formula-fed infants in two studies (one study found the opposite)^80^ and to be negatively associated with childhood asthma.^81^ Lastly, *Eubacterium hallii*, reclassified as *Anaerobutyricum soehngenii*, ^82^ was lower in the SSC group. *Anaerobutyricum soehngenii* can produce butyrate by utilizing the byproducts of other early colonizers that metabolize human milk oligosaccharides.^83,84^ It has been positively associated with infant colic^85^ and is under investigation for its benefits in relation to glucose metabolism.^86^

### Summary and conclusion

We found evidence that SSC affected microbiota composition in early infancy (2 and 5 weeks) and development in early and late infancy, as measured by volatility and microbiota age. Differential abundance and microbiota age analyses highlighted individual genera that differed between the groups. We proposed three mechanisms of action through which SSC may affect the microbiota. First, the transmission route appeared least relevant to the observed effects. Skin bacteria such as *Staphylococcus* were not differentially abundant between the groups. Also, the groups did not yet differ in the number of exclusively breastfed infants at five weeks (80.2% vs. 78.4%). Differences in breastfeeding were observed only at later ages. Thus, we can assume that physical contact, which occurs naturally through maternal caregiving and breastfeeding, was sufficient to transmit bacteria that were also transmitted through increased direct physical contact by the SSC intervention. Furthermore, previous research has demonstrated that most skin bacteria obtained from the mother are mainly detectable shortly after delivery but not at later time points^55^ as they do not colonize the gut. Second, our results suggested that breastfeeding may be one of the mechanisms by which the SSC intervention altered microbiota maturation long after the intervention, at one year of age. Third, considering prior evidence^56^ and the lack of differences in breastfeeding in early infancy, we hypothesize that the effect of SSC on volatility in early infancy may be due to its de-stressing effect.^17–19^ Future longitudinal studies would need to measure stress in addition to the measures we collected in order to investigate the proposed mechanism of SSC influencing microbiota volatility by acting as a stress buffer.

This preregistered study is the first RCT to assess the effects of SSC on the developing gut microbiota. Strengths include a randomized controlled design with blind recruitment and a low dropout rate throughout the intervention phase. In addition, microbiota and relevant covariates were sampled at several time points and included a follow-up microbiota sample long after the intervention was finalized. This allowed us to estimate the effect of SSC on microbiota development and explore one possible mechanism. Furthermore, we investigated a broad set of features of the microbiota and took measures to ensure the robustness of the results. Our analyses revealed that it is important to look beyond alpha diversity, beta diversity, and differential abundance analysis and include features that reflect microbiota development (volatility and microbiota age). Our study has some limitations. Since we did not measure all (time-varying) variables that influence protocol adherence, we may not exclude potential confounding bias in the per-protocol analysis estimates.^87,88^ Note however, this limitation is not relevant for our intention-to-treat analysis, which is regarded as the preferred analytic approach for RCTs. Finally, the generalizability of the study is limited, given the relatively homogeneous sample with mainly families of highly educated mothers.

In conclusion, we provide evidence that a postpartum skin-to-skin intervention in full-term infants may influence gut microbiota composition and volatility in early infancy, as well as microbiota age (for chronological age) in late infancy. The effects on microbiota age may have been partially mediated by SSC prolonging breastfeeding duration. It is highly desirable to replicate these findings to validate their robustness and establish their generalizability. Future studies would benefit by extending microbiota sampling beyond the first year of life to investigate the effect of SSC on microbiota maturation at later time points. Including variables that measure stress would allow for the investigation of whether the intervention has an effect on microbiota volatility by reducing stress.

## Methods

### Study design

This RCT included two parallel groups: an intervention group and a passive control.^38^ Ethics approval was granted by the ethics committee of the Social Science Faculty of Radboud University (ECSW2015–2311–358). This study was registered in the Netherlands Trial Register (NTR5697). It followed CONSORT guidelines and the protocol was published.^38^

### Participants

Expectant mothers from the Nijmegen region were recruited between April 2016 and September 2017. Recruitment was performed with the help of a database of pregnant mothers who expressed interest in participating in scientific research, as well as via promotions at pregnancy clubs, baby fairs, and baby shops. Prenatal characteristics were examined during the last trimester of pregnancy, using an eligibility survey. Expectant women were eligible to participate when they were at least 18 years old, had good physical and mental health, had a singleton pregnancy, were not using drugs during pregnancy, and had sufficient understanding of the Dutch language. After the birth of their child, mothers were excluded if their child was born before 37 weeks, had congenital anomalies, a birth weight less than 2500 g, and/or a 5-minutes Apgar score of < 7.

### Procedure

#### Prenatal

Detailed study information was provided to eligible women during a home visit between weeks 34–36 of gestation. After informed consent was obtained, only mothers allocated to the SSC group were encouraged to engage in at least one uninterrupted daily hour of SSC, starting immediately after birth until including the fifth postnatal week (Dutch mothers are entitled to 10 to 12 weeks of paid leave after birth). Detailed written and oral instructions about the SSC protocol, optimal SSC position and safety were provided. CAU mothers received no additional information, and all mothers were encouraged to contact the principal investigator when experiencing any problems during the study by phone, text message or e-mail. Besides SSC, both conditions underwent the same procedures.

#### Postnatal

All mothers reported daily information on SSC, holding (clothed physical contact), and no-contact in 15-min intervals every 2-3 h in a logbook throughout the 5-week intervention period. All mothers were contacted weekly by telephone (postnatal day 5 and 13) or text-message/e-mail (postnatal day 21 and 28) to remind mothers to complete the logbook, ask for questions/comments and, for SSC mothers, to discuss SSC obstacles. When their child was two weeks, five weeks and one year of age, parents were instructed to collect a fecal sample. The feces were collected directly from the first diaper that day, with the help of a provided plastic scoop. Parents were instructed to avoid any contact with surfaces or humans and put two or three scoops into a sterilized plastic tube. In addition to that, a stool questionnaire was completed, providing information on date of collection and infant health. After being temporarily stored in the home freezer (−20 degrees Celsius), the samples were collected during the home visits at 5 weeks and 1 year and then stored at −80 degrees Celsius. During the same home visits, questionnaires were obtained with information on covariates, such as gestational age, birth mode and feeding patterns.

### DNA extraction and processing to microbiota features

The samples were transported to the Laboratory of Microbiology at Wageningen University and stored at −80°C until they were processed for DNA extraction as published previously^89^ and described in the following: DNA extraction was carried out with the Maxwell 16 TOTAL RNA system (Promega, Wisconsin, USA) in conjunction with Stool Transport and Recovery Buffer (STAR; Roche Diagnostics Corporation, Indianapolis, IN). The V4 region of the 16S ribosomal RNA (rRNA) gene was amplified in duplication, generating amplicons of approximately 290bp. PCR reactions comprised 0.5 µl Phusion Green Hot Start II High-Fidelity DNA polymerase (Thermo Scientific, US) at 2 U/µl, 1 µl of 10um barcoded primers 515F-n(5’-GTGYCAGCMGCCGCGGTAA-3’) and 806 R-n(5’- GGACTACNVGGGTWTCTAAT-3’), 10 µl 5×Phusion Green HF Buffer (Thermo Scientific, US), 1 µl of 10 mM dNTPs mix (Promega Corporation, US), 36.5 µl Nuclease-free water, and 1 µl of 20ng/µl DNA template. PCR involved an initial denaturation period of 30s at 98°C, followed by 25 cycles of denaturation (98°C, 10 s), annealing (50°C, 10 s), and extension (72°C, 10 s), concluding with a final elongation step (72°C, 7 min). PCR products were validated through gel electrophoresis and purified using the HighPrep® PCR kit (MagBio Genomics, Alphen aan den Rijn, Netherlands). DNA concentration was assessed using a fluorometer (DS-11; DeNovix) with the Qubit® dsDNA BR Assay Kit (Life Technologies, Leusden, Netherlands). Barcoded samples belonging to the same library (200 ng) were combined. Each library incorporated 69 unique barcode tags, with 2 specifically designed for artificial control communities representing human gut microbiota. The mixture underwent purification again to achieve a final volume of 40 µl using the HighPrep® PCR kit. Sequencing was performed on the Illumina platform at Eurofins Genomics in Germany. Data were pre-processed from raw genetic sequences to amplicon sequence variant (ASV) tables using the NGTax2 pipeline (version 2.1.74) with the SILVA database (version 138.1). The sequence data, together with the metadata, were stored in a TreeSummerizedExperiment (TreeSE) container^90^ for further analysis. To analyze whether the gut microbiota differed between the SSC and CAU groups, the different features of the infant gut microbiome were evaluated. For alpha diversity, we calculated Shannon, inverse Simpson, Faith, and Chao1 indices. For beta diversity, we calculated the Aitchison distance and Bray – Curtis similarity. For volatility, we calculated the Aitchison distance sequentially between intra-individual samples.^56^ The microbiota age was determined as described by Subramanian et al.^60^ and we controlled for age in the regression models. We used samples from other longitudinal studies (BIBO and BINGO) to train the Random Forest model. Functional pathways, specifically the gut-brain-module (GBM), were calculated as described by Bastiaanssen et al.^59^

### Statistical analysis

In the following section, we briefly describe the statistical analysis. For a more detailed description see the preregistration (https://doi.org/10.17605/OSF.IO/S45MU). All analyses were performed in R (version 4.2.1)^91^ and the code was published (https://doi.org/10.5281/zenodo.8155205). Missing covariates and outcome variables were imputed using predictive mean matching (*m* = 50)^92^ with the package *mice* in R.^93^ Deviations of results from complete case analyses were to be reported. For each feature of the gut microbiota (alpha diversity, beta diversity, genus level abundance, volatility, microbiota age, and GBM), we performed intention-to-treat (ITT) and per-protocol (PP) analyses. We used Bayesian robust linear models to regress alpha diversity, volatility, and microbiota age on SSC and the covariates. These models were fit using the *brms* package^94^ with default priors and a Student’s t distribution for the response variable. For differential abundance analysis (genus level and GBMs), we utilized ANCOMBC,^95^ LinDA,^96^ and MaAsLin2.^97^ Combining the three methods ensures robustness of the presented findings. Furthermore, we applied a false-discovery rate (FDR) of FDR < 0.2 to control false discoveries while also limiting false negatives. The *adonis2* function from the *vegan* package was used for beta diversity analyses.^98^ We accounted for non-independence by specifying random intercepts in models that included repeated samples of an individual. Different alpha diversity indices were calculated using the R package mia.^99^

### Covariates

In the ITT analyses, we can assume that randomization prevented confounding of the average causal effect estimate (Figure S9). Therefore, we only added covariates that may improve the precision of our estimate of interest in a data-driven manner.^100^ Possible mediators of the effect of SSC, such as breastfeeding, had to be excluded to estimate the total effect of SSC.^100^ Breastfeeding was only added to determine the magnitude of the direct effect of SSC on the microbiota once an effect of SSC was detected. If the direct effect is smaller than the total effect upon inclusion, while SSC is positively related to breastfeeding and breastfeeding is related to the microbiota, this would suggest a mediating role of breastfeeding under the assumed directed acyclic graph (Figure S9).^100^ For the Bayesian models, we used leave-one-out cross-validation^101^ to evaluate whether any of the following variables improved model fit per microbiome feature: C-section, birth weight, siblings (yes/no), sex, Apgar (at 5 minutes), gestational age at birth, and education level. To determine whether SSC had an effect across all samples and over time, we modeled SSC in interaction with age across the models that included the samples obtained at 2 and 5 weeks. When modeling the samples at 52 weeks, we omitted the interaction term. For the PP analyses, we did not use the data-driven approach because confounding of the total effect estimate is possible, and we needed to make stronger assumptions.^87,88^ Measured variables that may influence whether an individual actually received the treatment (irrespective of assigned treatment) would need to be included to avoid confounding. Here, we included the following additional covariates in the models: birth weight, gestational age at birth, education level, cesarean section, and sex. Sensitivity analyses were performed using complete case analyses, and all covariates were excluded from the ITT analyses. The results did not differ meaningfully unless reported otherwise.

## Supplementary Material

Supplemental Material

## Data Availability

The research data are part of an ongoing study but can be requested from C de Weerth (Carolina.deWeerth@radboudumc.nl) for scientific purposes.
