# Supplementary material for: Daily skin-to-skin contact alters microbiota development in healthy full-term infants
Source: Gut Microbes. 2024 Jan 10;16(1):2295403. doi: 10.1080/19490976.2023.2295403 (PMC10793693; doi:10.1080/19490976.2023.2295403)
Supplement: Supplemental Material [file KGMI_A_2295403_SM3931.pdf]

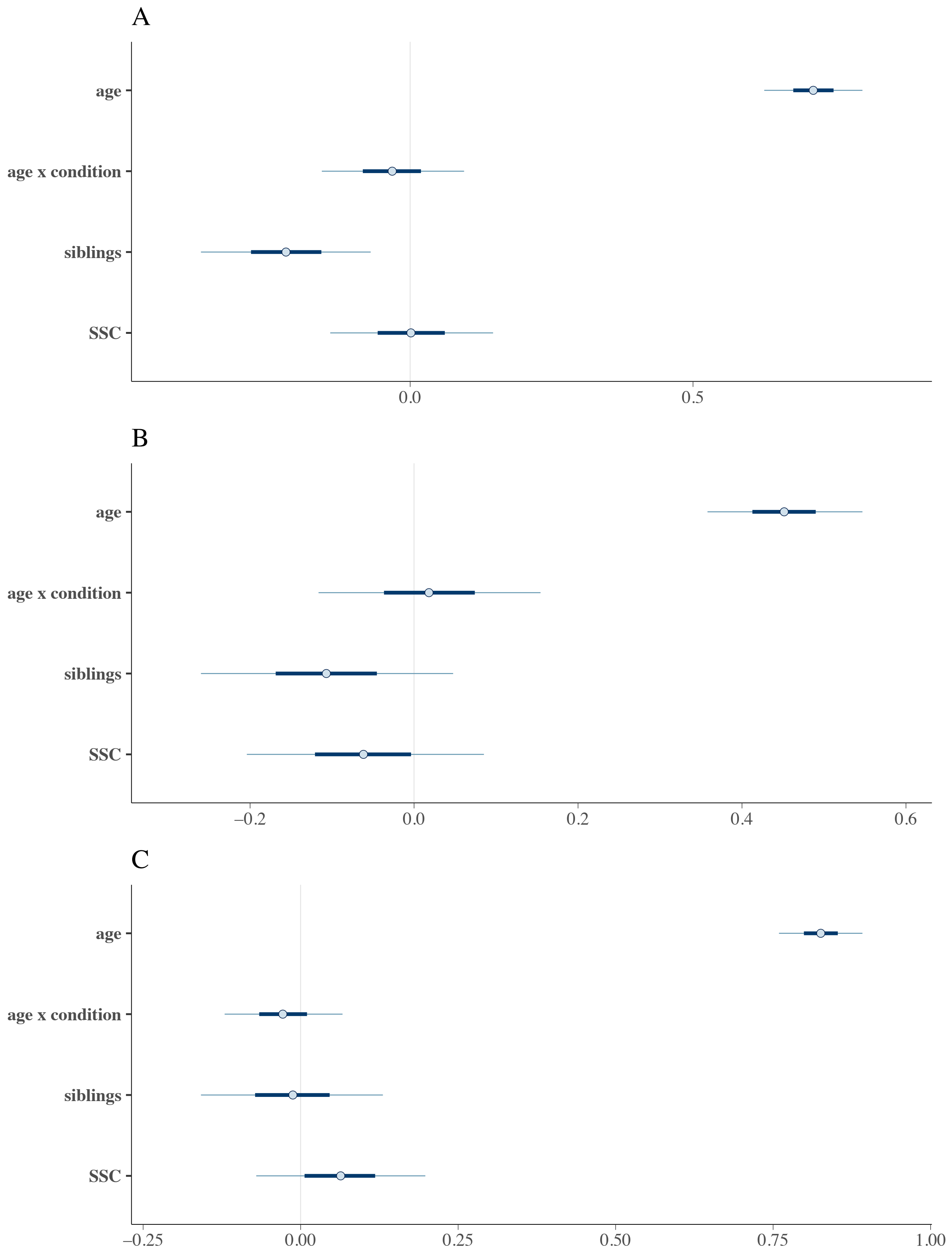

Figure 1: Posterior distributions (mean, 50% and 95% of the probability mass) of beta coefficients for the alpha diversity models (intention-to-treat analysis) using Shannon (A), Chao1 (B) or Faith (C) index.

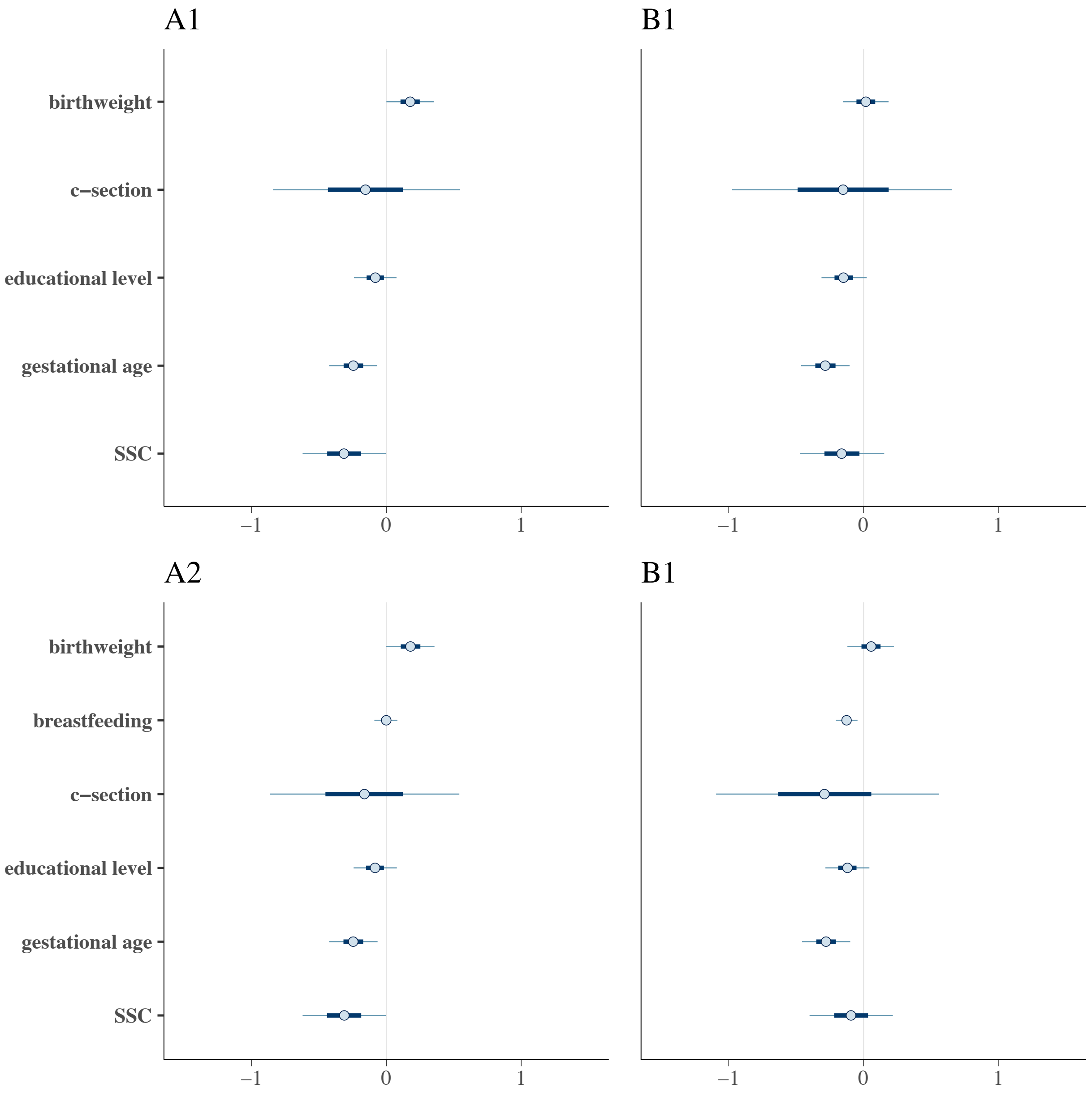

Figure 2: Posterior distributions (mean, 50% and 95% of the probability mass) of beta coefficients for the volatility models (intention-to-treat analysis) using volatility between 2 and 5 weeks (A) and 5 and 52 weeks (B) without (1) and with (2) breastfeeding.

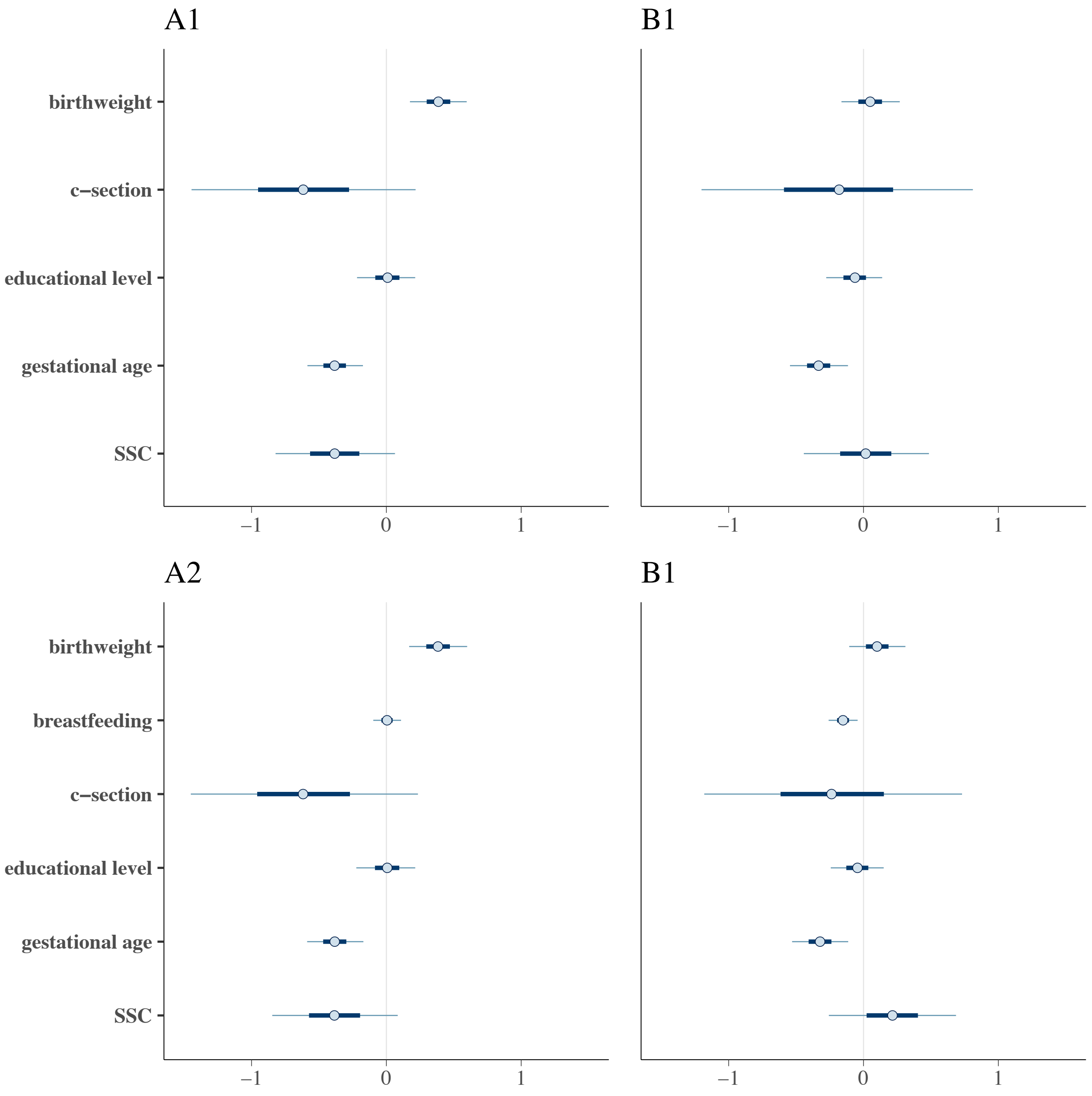

Figure 3: Posterior distributions (mean, 50% and 95% of the probability mass) of beta coefficients for the volatility models (per-protocol analysis) using volatility between 2 and 5 weeks (A) and 5 and 52 weeks (B) without (1) and with (2) breastfeeding.

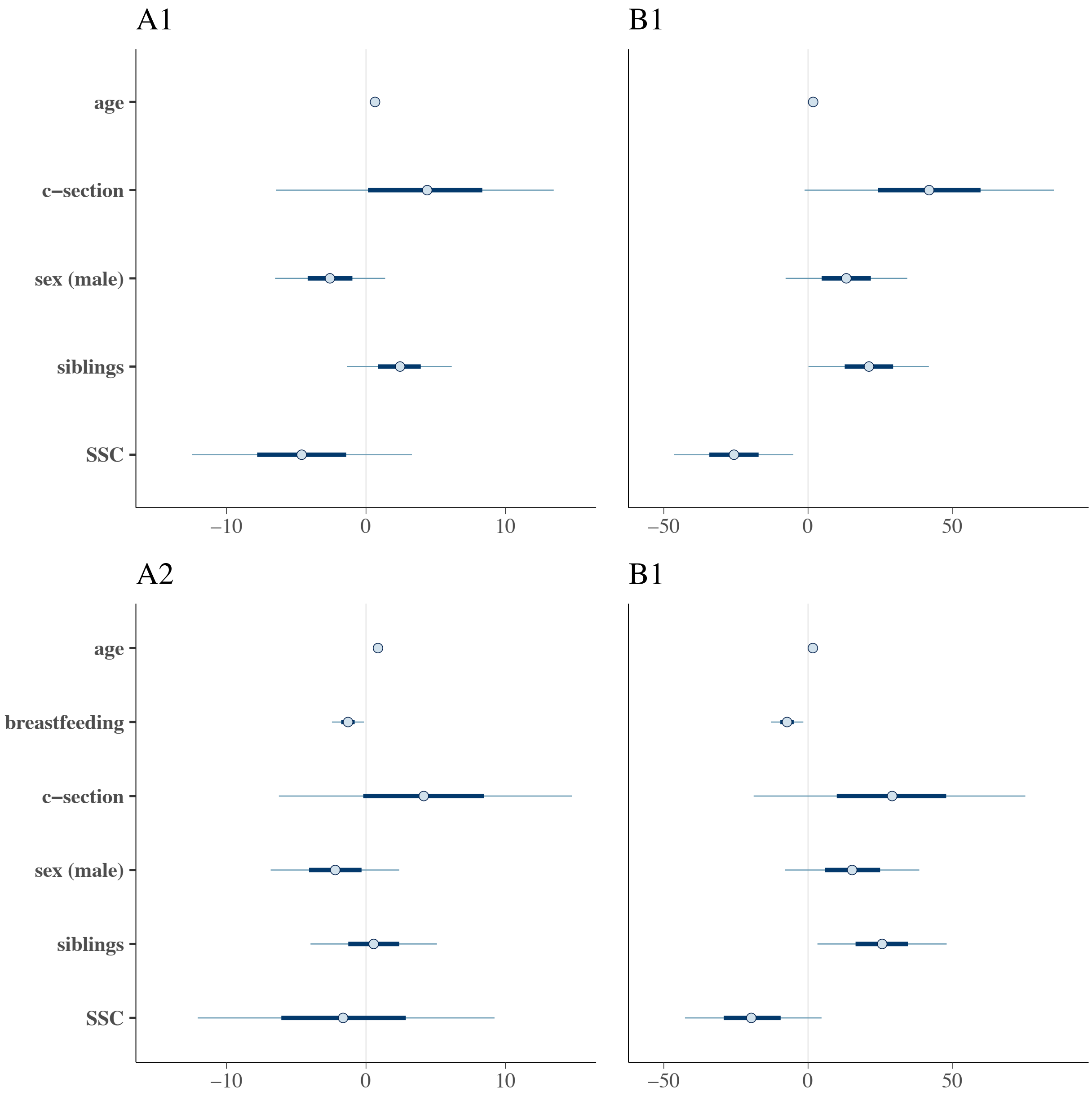

Figure 4: Posterior distributions (mean, 50% and 95% of the probability mass) of beta coefficients for the microbiota age models (intention-to-treat analysis) using microbiota age scores in early (A) and late infancy (B) without (1) and with (2) breastfeeding.

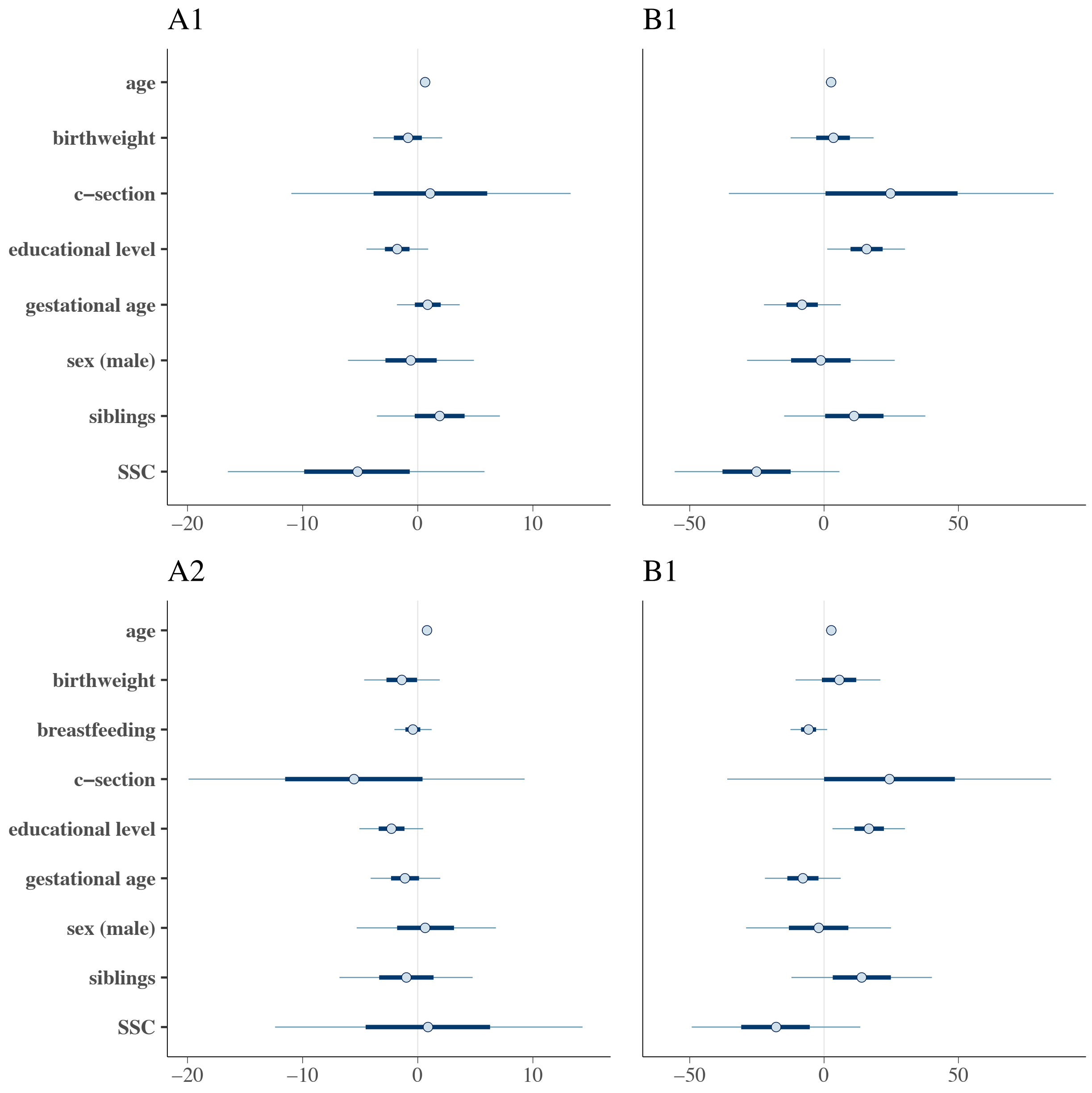

Figure 5: Posterior distributions (mean, 50% and 95% of the probability mass) of beta coefficients for the microbiota age models (per-protocol analysis) using microbiota age scores in early (A) and late infancy (B) without (1) and with (2) breastfeeding.

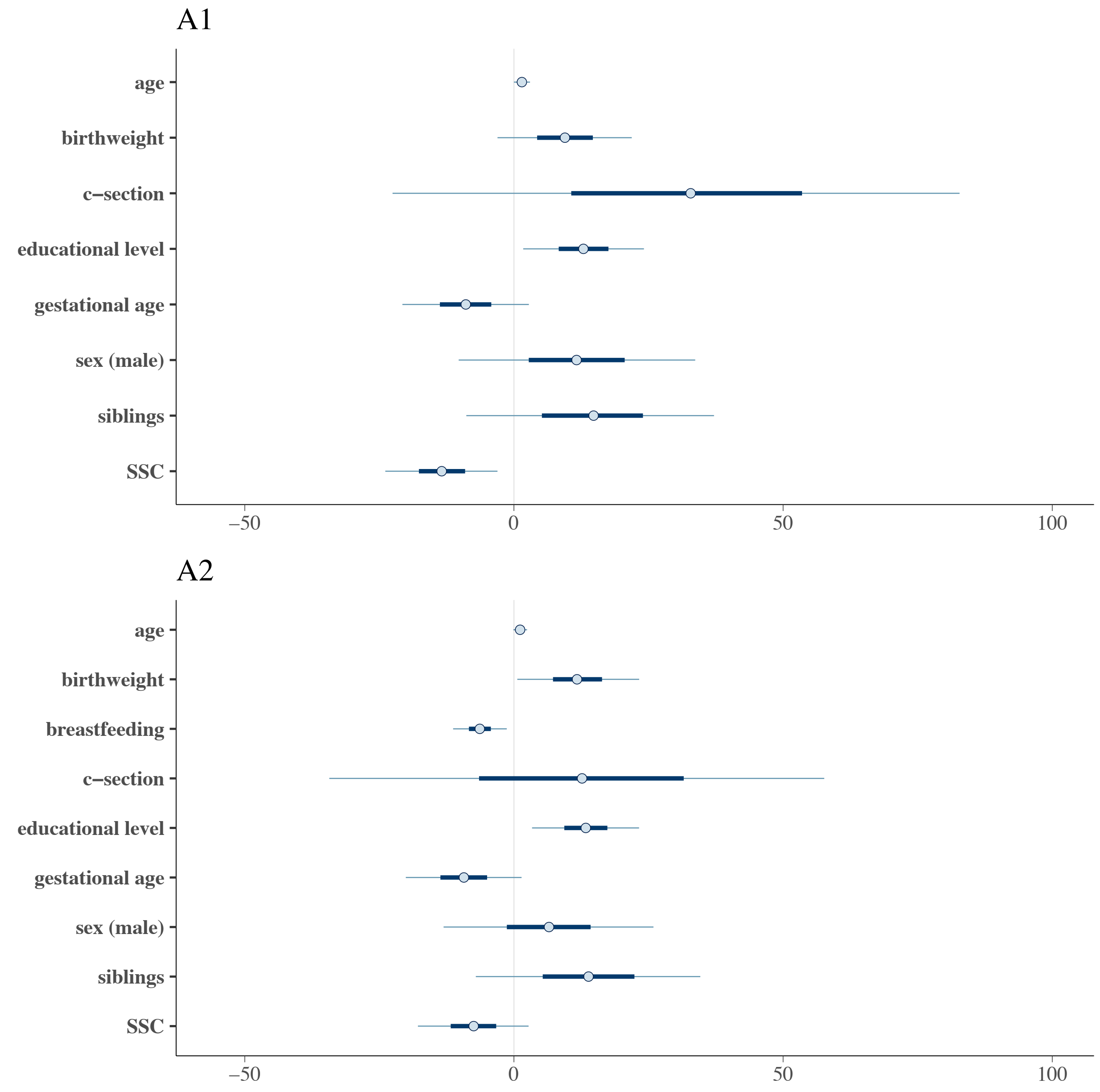

Figure 6: Posterior distributions (mean, 50% and 95% of the probability mass) of beta coefficients for the microbiota age models (dose-response analysis) using microbiota age scores in late infancy without (1) and with (2) breastfeeding.

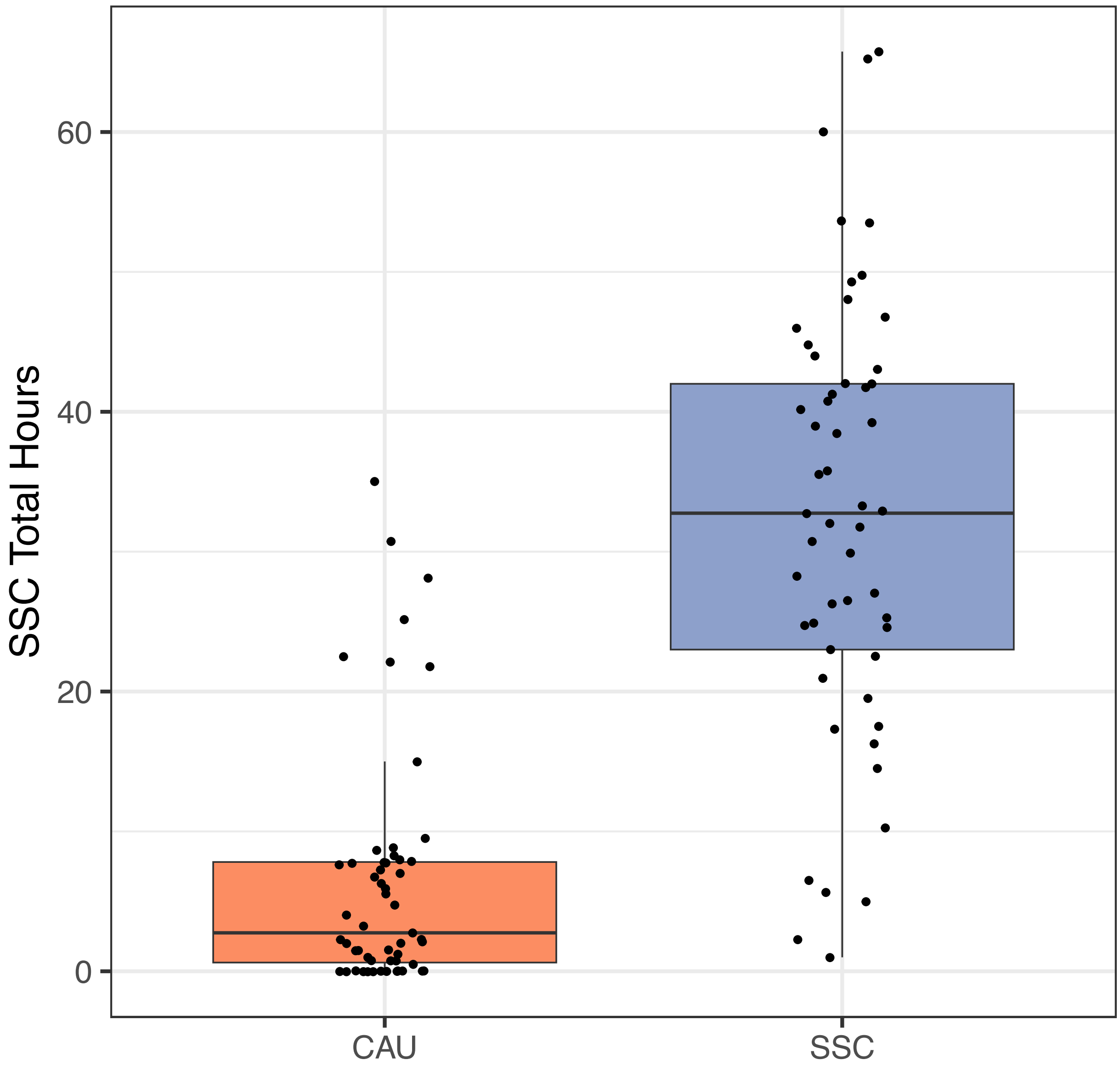

Figure 7: Boxplots of the number of provided skin-to-skin hours between groups.

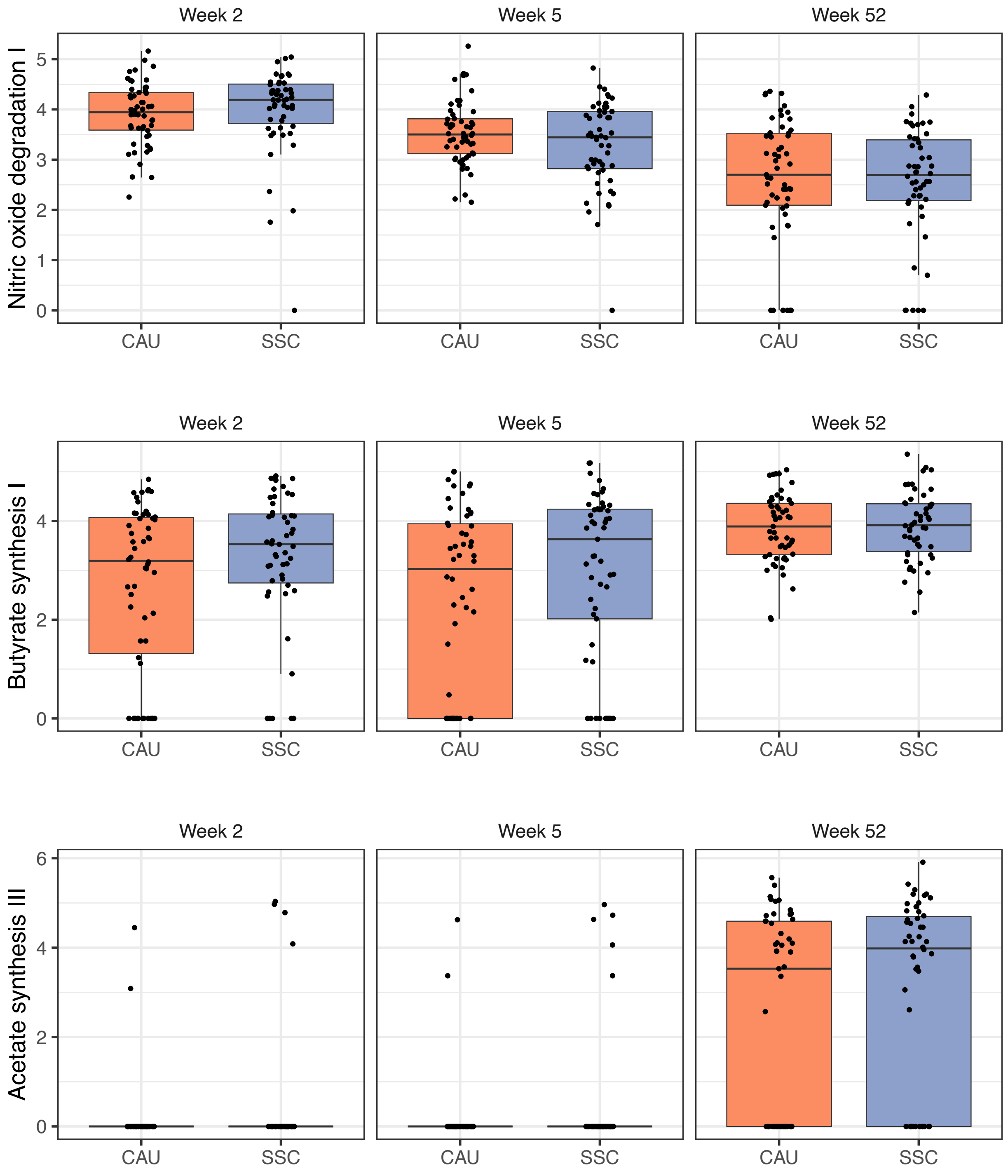

Figure 8: Boxplots of gut-brain-modules that were differentially abundant between SSC (blue) and CAU (red).

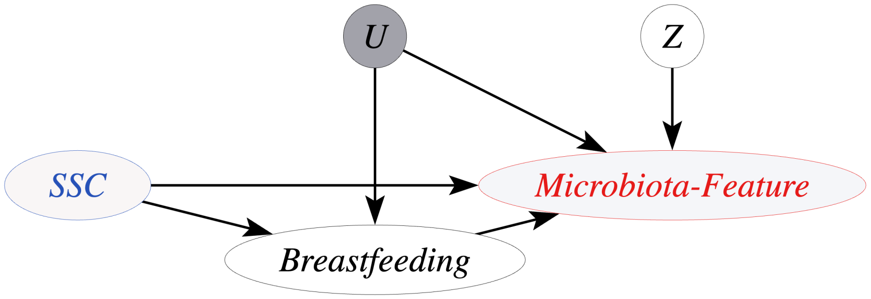

Figure 9: Directed acyclic graph depicting assumptions for the statistical analyses. U represents unmeasured variables. Z reflects all the measured variables that are known to influence the gut microbiota that are added in a data-driven approach in some of our models as they may improve precision of our estimate of interest (see covariate section in the methods and Cinelli et al. (2020). Note that we performed sensitivity analysis by leaving out any variables Z. Given the randomization of participants into skin-to-skin (SSC) or care-as-usual, there are no arrows pointing towards SSC and the total effect estimate of SSC on the microbiota is assumed to be unbiased in contrast to any other potential effects that may be confounded by U.
